# Routinely detected indicators in plasma have a predictive effect on the identification of HIV-infected patients with non-tuberculous mycobacterial and tuberculous infections

**DOI:** 10.1186/s40249-017-0347-6

**Published:** 2017-11-02

**Authors:** Ren-tian Cai, Feng-xue Yu, Zhen Tao, Xue-qin Qian, Jun Chen, Hong-zhou Lu

**Affiliations:** 1 Department of Infectious Diseases, Nanjing First Hospital, Nanjing Medical University, Nanjing, China; 2 Department of Infectious Diseases, Shanghai Public Health Clinical Center, Fudan University, Shanghai, China; 3 Department of Nephrology, the Second Affiliated Hospital of the Southeast University, Nanjing, China; 4 Department of Mycobacteria Culture, Shanghai Public Health Clinical Center, Fudan University, Shanghai, China; 5 Huashan Hospital affiliated to Fudan University, Shanghai, China; 6 Medical College of Fudan University, Shanghai, China; 7 2901 Caolang road, Shanghai, 201508 China

**Keywords:** HIV, Tuberculosis, Non-tuberculous mycobacteria

## Abstract

**Background:**

It is difficult to quickly distinguish non-tuberculous mycobacterial (NTM) infection from tuberculosis (TB) infection in human immunodeficiency virus (HIV)-infected patients because of many similarities between these diseases. A simple and effective way to determine the differences using routine blood tests is necessary in developing countries.

**Methods:**

A retrospective cohort study was conducted to recruit HIV-infected patients with either NTM infection or TB infection diagnosed for the first time according to mycobacterial culture and microscopic identification from May 2010 to March 2016. These data included the analysis of blood cells, liver function, renal function, C-reactive protein (CRP), and erythrocyte sedimentation rate (ESR), and were compared between the HIV/TB and HIV/NTM groups.

**Results:**

A total of 240 patients were enrolled. The number of HIV/TB and HIV/NTM patients was 113 and 127, respectively. There were no significant differences in the CD4 T-cell count, age, sex, percentage of patients initiating antiretroviral therapy (ART) before the explicit diagnosis of TB or NTM infection. NTM infection was more likely to be restricted in the pulmonary while TB infection also involves extra-pulmonary sites. Both the leukocyte count(5.60 × 10^9^/L) and the proportion of neutrophils in the leukocyte count (76.70%) in the HIV/TB group were significantly higher than those in the HIV/NTM group (4.40 × 10^9^/L [*P* = 0.0014] and 69.30% [*P* < 0.001]. The analysis of liver function markers indicated that the concentration of albumin but not ALT and AST was significantly lower in the HIV/TB group than in the HIV/NTM group (*P* < 0.001). The creatinine and urea levels were not significantly different between the two groups. The ESR (84.00 mm/h) and the concentration of CRP (59.60 mg/L) were significantly higher in the HIV/TB group than in the HIV/NTM group (52.00 mm/h and 19.60 mg/L, respectively) (*P* < 0.001). To distinguish TB infection from NTM infection, the best cut-off value was 69.5 mm/h for ESR, with a positive predictive value (PPV) of 0.740 and negative predictive value (NPV) of 0.721, and 48.8 mg/L for CRP, with a PPV of 0.676 and NPV of 0.697.

**Conclusion:**

The dissemination character as well as stronger immune response characterized by higher inflammation markers (e.g. WBC, ESR, CRP) can help distinguish TB from NTM infection in HIV-infected patients who need empirical therapy or diagnostic therapy immediately in low-income areas.

**Electronic supplementary material:**

The online version of this article (doi:10.1186/s40249-017-0347-6) contains supplementary material, which is available to authorized users.

## Multilingual abstract

Please see Additional flie 1 translations of the abstracts into the six official working languages of the United Nations.

## Background

Human immunodeficiency virus (HIV)-infected patients, particularly those with acquired immune deficiency syndrome (AIDS), are commonly infected with several pathogenic microorganisms. Mycobacteria, including non-tuberculous mycobacteria (NTM) and mycobacterium tuberculosis (TB), are an important group of pathogens that infect HIV/AIDS patients more often than immunocompetent individuals [1–4]. The regimens and course of TB/NTM infection treatment are different. Anti-TB drug regimens include isoniazid, rifampicin, pyrazinamide, and ethambutol, etc. Drug regimens for NTM infections include kanamycin and rifabutin, or azithromycin and rifabutin [5, 6]. Although rifabutin has anti-TB action, the treatment of patients infected with TB or NTM is not directly analogous [5–7]. Both HIV/TB and HIV/NTM-infected patients may die if they are not treated promptly and accurately.

However, the clinical manifestations of NTM/HIV and TB/HIV are similar, including fever, cough, and fatigue [8, 9]. Therefore, it is difficult to distinguish the type of mycobacterial infection by the patient’s clinical status. The golden evidence is mycobacterial culture and microscopic identification of the bacterial strains. However, it takes 3 to 4 weeks to obtain the results of a liquid culture medium for mycobacteria [10], and the positivity rate of mycobacterial culture is low (less than 50%) [10, 11]. The MPB64 antigen has been shown to be specific for the TB [12] and used clinically for identification of TB after mycobacterium culture [13, 14]. Nevertheless, this period is too long for patients who need anti-NTM or anti-TB therapies.

Other immunological indicators such as interferon gamma release assays (IGRAs) can rapidly diagnose TB infection. However, the sensitivity and specificity of IGRAs are compromised in HIV-infected patients [15, 16] and these methods cannot distinguish latent from active TB infection [17]. Second, several NTM (e.g. in *Mycobacterium kansasii*, *M. szulgai*, and *M. marinum*) also express the ESAT-6 and CFP-10 protein, which is detected by IGRAs [18]. Furthermore, these immunological methods are expensive and difficult to implement in low-income countries with a high burden of infectious diseases, including HIV, TB, and NTM.

Therefore, it is necessary to implement a simple and effective strategy to distinguish NTM from TB infection in HIV infected patients in low-income area who need empirical therapy or diagnostic therapy immediately. The purpose of this study was to help chronic HIV-infected patients suspected with TB/NTM infection distinguish TB from NTM by comparing the levels of routinely detected indicators in blood.

## Methods

### Study design

**Fig. 1:**
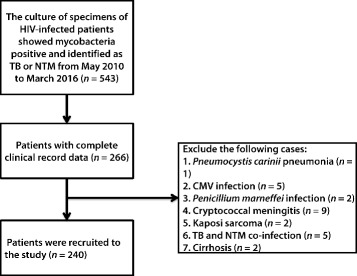
Flow diagram of the study

Mycobacterial culture specimens were collected from May 2010 to March 2016 in the Department of Mycobacterial Culture in Shanghai Public Health Clinical Center, the largest referral center for patients with HIV infection or AIDS in East China. The diagnosis of TB or NTM infection was based on the positive culture of *Mycobacterium* in at least one sample from the sputum, urine, blood, bone marrow, and/or cerebrospinal fluid. The mycobacteria were identified as TB or NTM by the immune colloidal gold technique. A total of 543 samples was collected. The clinical data of patients were obtained based on the information provided by specimen data. Then, 266 patients were diagnosed with active tuberculosis or NTM infection for the first time. Blood cells, liver function, renal function, C-reactive protein (CRP), and erythrocyte sedimentation rate (ESR) were analyzed. Data were collected before the initiation of anti-TB or anti-NTM therapies. The patients with illnesses that might affect the tested indicators, including sepsis, HBV or HCV infection, cirrhosis, liver and/or kidney failure were excluded. The patients co-infected with both TB and NTM (*n* = 5) and those with the following AIDS-related opportunistic infections and malignancies or other internal diseases were excluded: *Pneumocystis jiroveci* pneumonia (*n* = 1), cytomegalovirus infection (*n* = 5), *Talaromyces marneffei* infection (*n* = 2), cryptococcal meningitis (*n* = 9), Kaposi sarcoma (*n* = 2), and cirrhosis (*n* = 2) (Fig. 1). The remaining 240 patients met the requirements of the study and were divided into two groups: HIV/TB and HIV/NTM.

### Detection of routine indicators

Complete blood count was carried out using automated blood cell analyser. The output results included leukocyte count, neutrophils, lymphocytes percentage and haemoglobin (Hb). The liver function tests including alanine aminotransferase (ALT) aspartate amino trasferase (AST) and renal function tests including urea, creatinine and serum albumin were measured by colorimetry using automated analyser. CRP was tested by turbidimetric inhibition immunoassay and ESR was detected by Westergren method.

### Statistical analysis

Data normality was assessed using the Shapiro-Wilk test. Normally distributed data are shown as the mean ± standard deviation, whereas non-normally distributed data are shown as the median and interquartile range (IQR). The Levene’s test was used to evaluate the variance homogeneity of the data. Student’s *t*-test was conducted to assess differences between the two groups in cases in which the data showed normal distribution and homogeneity of variance. The Wilcoxon rank-sum test was used to evaluate data with non-normal distribution or heterogeneity of variance. The chi-square (χ^2^) test was applied to analyze the categorical variables. The sensitivity and specificity of indicators significant differences were obtained by receiver operating characteristic (ROC) curve. The results were considered significant when the *P*-values were equal to or smaller than 0.05. All statistical analyses were conducted using IBM SPSS software version 19.0 (IBM SPSS, Inc., Armonk, NY, USA) and GraphPad Prism 6.0 (GraphPad Software Inc., San Diego, CA, USA).

## Results

### Characteristics of the study population

The clinical characteristics of the 240 patients enrolled are shown in Table 1.

**Table 1:** General characteristics of the study subjects

Variable		TB	NTM	*P-*value
Subjects	*n*	113	127	
Sex	Male	98	115	0.349
	Female	15	12	
Age	(years)	41(30.5–52.5)	41(32–52)	0.707
Onset at diagnosis	(days)	60(45–90)	60 (40–90)	0.435
TB or NTM infection	Pulmonary	64	106	<0.001
	Extrapulmonary	15	6	
	Pulmonary and extrapulmonary	34	15	
CD4 T cell count	Median (IQR)	40 (21–85)	38 (11–121)	0.744
(cells/μl)	< 50	65 (57.5%)	71 (55.9%)	0.896
	≥ 50	48 (42.5%)	56 (44.1%)	
Antiretroviral therapy	YES	42	55	0.333
	NO	71	72	

A total of 113 and 127 patients were diagnosed of HIV/TB and HIV/NTM co-infection, respectively. There were no significant differences in the age and sex between the two groups. The CD4 T-cell count and the distribution of CD4 T cells (< 50 cells/μl and ≥50 cells/μl) in the TB group was not significantly different from that in the HIV/NTM group. In addition, there was no significant intergroup difference in the percentage of patients who initiated ART before the specific diagnosis of HIV/TB or HIV/NTM infection. However, as regard of the sites of infection, NTM infection was more likely to be restricted in the pulmonary while TB infection also involve extra-pulmonary sites (pulmonary infection [*n* = 64, 56.6%], extra-pulmonary infection [*n* = 15, 13.3%], and pulmonary and extra-pulmonary infection [*n* = 34, 30.1%] in HIV/TB infection group vs pulmonary infection [106, 83.5%], extra-pulmonary infection [6, 4.7%], and pulmonary and extra-pulmonary infection [15, 11.8%] in HIV/NTM infection group *P* < 0.001) (Tables 1, 2, and 3).

**Table 2:** Group and subgroup of patients with active tuberculosis

Group	Number	Subgroup	Number
Pulmonary TB	64	Ordinary pulmonary TB	50
		Blood disseminated pulmonary TB	14
Extra-pulmonary TB	15	Lymph node TB	4
		Tuberculous peritonitis	10
		Skin tuberculosis and tuberculous choroiditis	1
Pulmonary and extra-pulmonary TB	34	Pulmonary TB and tuberculous peritonitis	17
		Pulmonary TB and lymph node TB	9
		Pulmonary and intestinal TB	2
		Pulmonary and liver TB	1
		Pulmonary TB combined with tuberculous pleurisy and lymph node TB	2
		Pulmonary and brain TB	1
		Pulmonary TB combined with tuberculous meningitis and intestinal TB	1
		Pulmonary and bronchial gland TB	1

**Table 3:** Group and subgroup of patients with NTM infection

Group	Number	Subgroup	Number
Pulmonary NTM	106	Pulmonary NTM	96
		Blood disseminated pulmonary NTM	10
Extra-pulmonary NTM	6	Skin NTM	1
		Spinal NTM	1
		NTM spinal meningitis	1
		Intestinal NTM	2
		NTM peritonitis and intestinal NTM	1
Pulmonary and extra-pulmonary NTM	15	Pulmonary and intestinal NTM	4
		Pulmonary and lymph node NTM	3
		Pulmonary and skin NTM	2
		Pulmonary and stomach NTM	1
		Pulmonary and NTM meningitis	2
		Pulmonary NTM and NTM peritonitis	2
		Pulmonary and bone NTM	1

### Accessible blood index

#### Routine blood test

**Fig. 2:**
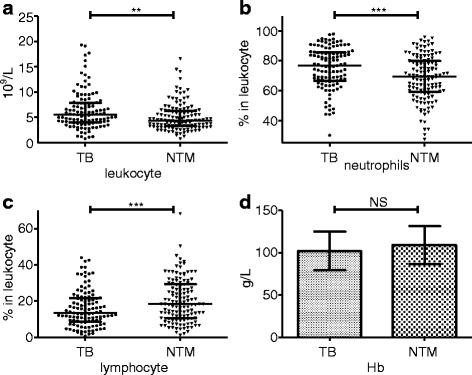
Routine blood test in TB and TNM patients with HIV infection. The detected indicators included leukocyte count (**a**), percentages of neutrophils in the leukocyte count (**b**), percentage of lymphocytes in the leukocyte count (**c**), and concentration of hemoglobin (Hb) (**d**). **: *P* < 0.01, ***: *P* < 0.001, NS: no significant difference

Both the leukocyte count (5.60 [4.00–7.85] × 10^9^/L) and the proportion of neutrophils (76.70% [66.50–85.60%]) in the HIV/TB group were significantly higher than those of the HIV/NTM group (4.40 [3.30–6.30] × 10^9^/L, *P* = 0.0014), 69.30%[59.10–80.10%], *P* < 0.001), respectively. However, the proportion of lymphocytes was significantly lower in the HIV/TB group (13.40% [8.55–21.75%]) compared with the HIV/NTM group (18.50% [10.50–29.30%], *P* < 0.001). No significant difference between the two groups in the concentration of hemoglobin (Hb) (Fig. 2).

#### Liver function

**Fig. 3:**
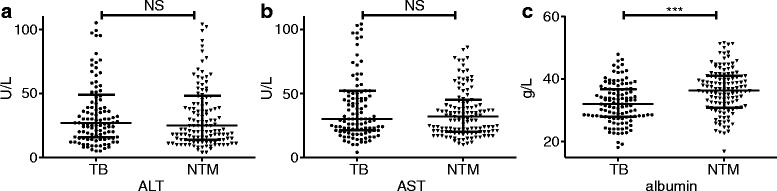
Liver function test in TB and TNM patients with HIV infection. The detected indicators included the concentration of alanine aminotransferase (ALT) (**a**), aspartate aminotransferase (AST) (**b**), and albumin (**c**). ***: *P* < 0.001, NS: no significant difference

We recorded three markers of hepatic function, alanine aminotransferase (ALT), aspartate aminotransferase (AST), and albumin. The concentration of albumin was significantly lower in the HIV/TB group than that of the HIV/NTM group. The levels of the two other markers were not significantly different between the two groups (Fig. 3).

#### Kidney function

**Fig. 4:**
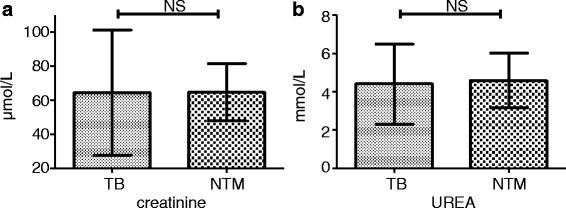
Kidney function test in TB and TNM patients with HIV infection. The detected indicators included creatinine (**a**), and urea nitrogen (UREA) (**b**). NS: no significant difference

The concentrations of the two indicators of kidney function (creatinine and urea nitrogen) were not significantly different between the two groups (Fig. 4).

#### ESR and CRP

**Fig. 5:**
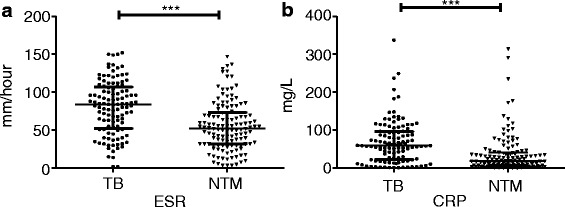
The ESR and CRP test in TB and TNM patients with HIV infection. The detected indicators included the erythrocyte sedimentation rate (ESR) (**a**) and C-reaction protein (CRP) (**b**). ***: *P* < 0.001

ESR and CRP are important indicators of human inflammation. We found that the ESR in the HIV/TB group (84.00 [52.50–107.00] mm/h) was significantly higher than that in the HIV/NTM group (52.00 [32.00–73.00] mm/h). Furthermore, the concentration of CRP (59.60 [23.45–96.35] mg/L) was significantly higher in the HIV/TB group compared with the HIV/NTM group (19.60 [8.10–41.90] mg/L), (*P* < 0.001) (Fig. 5).

#### ESR and CRP had a moderate effect on the identification of TB from NTM co-infection with HIV

The areas under the ROC curve (AUC) of ESR and the concentration of CRP were 0.703 and 0.709, respectively. The best cut-off value was 69.5 mm/h for ESR, with a sensitivity of 0.646 and a specificity of 0.724, and positive predictive value (PPV) of 0.740 and negative predictive value (NPV) of 0.721, and 48.8 mg/L for CRP, with a sensitivity of 0.655 and a specificity of 0.795, and PPV of 0.676 and NPV of 0.697. Although the leukocyte count, proportion of neutrophils and lymphocytes in leukocytes, and concentration of albumin were significantly different between the HIV/TB and HIV/NTM-infected patients, their AUC values were 0.609–0.636 (Table 4).

**Table 4:** Sensitivity and specificity of test indicators for distinguishing TB from NTM infections

Indicator	AUC	Maximum Youden index	Best cut-off point	Sensitivity	Specificity
ESR	0.703	0.370	69.5 mm/h	0.646	0.724
CRP	0.709	0.450	48.8 mg/L	0.655	0.795
Leukocytes	0.620	0.198	5.55 × 10^9^/L	0.513	0.685
Neutrophils	0.636	0.229	65.05% in leukocytes	0.788	0.441
Lymphocytes	0.609	0.181	17.75% in leukocytes	0.535	0.646
Albumin	0.620	0.198	5.55 g/L	0.513	0.685

## Discussion

Our results indicated that the prevalence of NTM and TB infection is similar in advanced HIV infection. Similarly, other studies reported that more people were infected with NTM than before [19, 20]. This result suggests that HIV-infected individuals may be infected with *Mycobacterium* and that NTM infections are more likely to be diagnosed than TB infections. Methods of identification of NTM include mycobacterial culture [10, 11] and then are distinguished by specific antibodies against TB [12, 13], or other immunological methods [14–16]. However, these take a long time to achieve definite results or are expensive and difficult to implement in low-income countries. Our results revealed that it was possible to identify co-infection with HIV and either NTM or TB by markers that are routinely detected in blood.

We found NTM infection was more probably to be located in the pulmonary but TB infection was more distributed in other tissues and organs including peritoneum, lymph nodes, intestinal tract, liver, brain and bronchial gland. Our study confirmed that the most common clinical manifestation of NTM disease is lung disease, while lymphatic and skin/soft tissue involvement as well as disseminated disease are also reported [21, 22]. However, NTM infections of soft tissue, lymph node or bone are less prevalent [23]. In contrast, tuberculosis easily disseminate to other organizations. One of the reasons is that tuberculosis granuloma play an important role in expansion and dissemination of tuberculosis infection [24].

There was no significant difference in the CD4 T cell count between the two study groups, and this result might suggest that the immunity of HIV/NTM or HIV/TB infection was similar. Although the guidelines from the Center for Disease Control and Prevention (CDC) emphasize that HIV-infected patients with a CD4 T-cell count lower than 50 cells/μl are more susceptible to infection with NTM but not with TB [6]. We found that even in cases of which the CD4 cell count was higher than 100 cells/μl, the patients could still be infected with NTM. A similar result was found in previous studies [4, 8, 25, 26].

In line with the dissemination character of TB infection in HIV-infected patients, we found these patients also have higher inflammation markers. Both the leukocyte count and neutrophils percentage in the HIV/TB group were significantly higher than those in the HIV/NTM group. Several previous studies found signals from dead or dying granuloma macrophages infected by tuberculous recruit neutrophils and stimulate neutrophil proliferation, which then phagocytose infected macrophages [27, 28]. This may partially explain the phenomenon found in our study.

For the first time, this study showed that the ESR and the concentration of CRP were significantly higher in HIV/TB patients than in the HIV/NTM group. The ESR and CRP had a moderate effect on the identification of co-infection with HIV and either TB or NTM. The probable cause of this difference was that TB infection might induce a stronger inflammatory response than NTM infection. The CRP concentration in HIV(−) patients infected with TB was also reported to be higher than in non-HIV patients infected with NTM [29].

These results, combined with data on leukocyte count and neutrophils, allow us to speculate that HIV patients infected with TB might present a stronger inflammatory response than HIV patients infected with NTM. This result may be because of the characteristics of the two types of microorganisms. TB is not an opportunistic pathogen. The patients have to undergo anti-TB chemotherapy after the diagnosis of active TB infection. However, NTM are common opportunistic pathogens and their natural environment is larger than that of TB. Furthermore, NTM can survive in the soil, water, milk, food products, aerosols, and wild and domestic animals [30–32]. Individuals are easily exposed to NTM but do not present symptoms and do not need treatment, indicating that NTM infections are harmless to the human body in most situations [3, 23]. Therefore, the infectivity and pathogenicity of NTM are lower than those of TB [23, 33]. However, NTM is the name of a variety of non-tuberculous mycobacteria, and some of which may be higher pathogenic [23].

Our results indicated that the concentration of albumin in HIV/TB patients was lower than that in the HIV/NTM group. This may be a result of increased albumin consumption in HIV/TB group as they have stronger inflammation as compared with HIV/NTM group.

One of the limitations of our study was that the NTM was not classified into subtypes as they may have different characters. In addition, the retrospective nature of the current study also limited the degree to which these findings can apply to current practice.

## Conclusions

The dissemination character as well as stronger immune response characterized by higher inflammation markers (e.g. WBC, ESR, CRP) can help distinguish TB from NTM infection in HIV-infected patients who need empirical therapy or diagnostic therapy immediately.

## Additional file

Additional file 1: Multilingual abstract in six official working languages of the United Nations. (PDF 630 kb)
