# Correction to: Routinely detected indicators in plasma have a predictive effect on the identification of HIV-infected patients with non-tuberculous mycobacterial and tuberculous infections

**DOI:** 10.1186/s40249-017-0376-1

**Published:** 2017-11-23

**Authors:** Ren-tian Cai, Feng-xue Yu, Zhen Tao, Xue-qin Qian, Jun Chen, Hong-zhou Lu

**Affiliations:** 1 Department of Infectious Diseases, Nanjing First Hospital, Nanjing Medical University, Nanjing, China; 2 Department of Infectious Diseases, Shanghai Public Health Clinical Center, Fudan University, Shanghai, China; 3 Department of Nephrology, the Second Affiliated Hospital of the Southeast University, Nanjing, China; 4 Department of Mycobacteria Culture, Shanghai Public Health Clinical Center, Fudan University, Shanghai, China; 5 Huashan Hospital affiliated to Fudan University, Shanghai, China; 6 Medical College of Fudan University, Shanghai, China

## Correction

After publication of this article [1] it came to our attention that the affiliation of Jun Chen and Hong-zhou Lu were incorrectly shown.

Jun Chen’s affiliation should have been given as Department of Infectious Diseases, Shanghai Public Health Clinical Center, Fudan University, Shanghai, China.

Hong-zhou Lu should have been given as Department of Infectious Diseases, Shanghai Public Health Clinical Center, Fudan University, Shanghai, China. Huashan Hospital affiliated to Fudan University, Shanghai, China. Medical College of Fudan University, Shanghai, China.

The original article has been updated to reflect this change.
