# Supplementary material for: Routinely detected indicators in plasma have a predictive effect on the identification of HIV-infected patients with non-tuberculous mycobacterial and tuberculous infections
Source: Infect Dis Poverty. 2017 Nov 2;6:132. doi: 10.1186/s40249-017-0347-6 (PMC5667182; doi:10.1186/s40249-017-0347-6)
Supplement: Additional file 1: — Multilingual abstract in six official working languages of the United Nations. (PDF 630 kb) [file 40249_2017_347_MOESM1_ESM.pdf]

Translation of the abstract into the six official working languages of the United Nations

الكشف عن المؤشرات بشكل روتيني في البلازما له تأثير تنبئي على تحديد المرضى المصابين بفيروس نقص المناعة البشرية المقترن بعدوى غير سلية متفطرة أو بعدوى سلية

رن تيان تساى، فنج شيويه يو، تشن تاو، شيويه تشين تشيان، جون تشن، هونج تشو لو

ملخص

خلفية: من الصعب التمييز بسرعة بين العدوىالسلية غير المتفطرة و مرض السل في مرضى فيروس العوز المناعي البشري (HIV) بسبب العديد من أوجه التشابه بين هذين المرضين. وهناك حاجة لوجود طريقة بسيطة وفعالة لتحديد الاختلافات باستخدام اختبارات الدم الروتينية والذي يعد أمرا ضروريا في البلدان النامية.

أساليب: أجريت دراسة أترابية بأثر رجعي لفحص المرضى المصابين بفيروس العوز المناعي البشري المقترن بعدوى سلية متفطرة أو عدوى سل مشخصة لأول مرة استنادا إلى مزرعة متفطرة والتعرف المجهري من مايو 2010 إلى مارس 2016. البيانات هذه وشملت بين مقارنتها الحمراوتمت الدم كرات ترسيب ومعدل C، الارتكاسي البروتين الكلى، وظيفة الكبد، وظائف دم،الـ خلايا تحليل المتفطرة غير السلية العدوى / البشري المناعي العوز وفيروس السل / البشري المناعي العوز يروس مجموعاتالنتائج: وقد تم تسجيل ما مجموعه 240 مريضا. وكان عدد المصابين بفيروس العوز المناعي البشري / السل وفيروس العوز المناعي البشري / العدوى السلية غير المتفطرة 113 و 127 على التوالي. لم تكن هناك فروق ذات دلالة إحصائية في عدد الخلايا التائية CD4، والعمر، والجنس، ونسبة المرضى الذين يبدأون العلاج المضاد للفيروسات القهقرية قبل التشخيص الصريح للعدوى السلية أو السلية غير المتفطرة. وكان من المرجح أن تكون العدوى السلية غير المتفطرة مقيدة بالرئة في حين أن التهاب السل ينطوي أيضا على مواقع خارج الرئة. كل من عدد الكريات البيضاء (5.60 × 10^9/ لتر) ونسبة العدلات في عدد الكريات البيضاء (76.70٪) في مجموعة فيروس العوز المناعي البشري / السل كانت أعلى بكثير من تلك الموجودة في مجموعة فيروس العوز المناعي البشري / العدوى السلية غير المتفطرة (4.40 × 10^9/ لتر [P= 0.0014] و 69.30٪ [0.001>P]. وأشار تحليل مؤشرات وظائف الكبد إلى أن تركيز الألبومين ولكن ليس ناقلة أمين الألانين وناقلة أمين الأسبارتات كان أقل بكثير في مجموعة فيروس العوز المناعي البشري / السل عن مجموعة فيروس العوز المناعي البشري / العدوى السلية غير المتفطرة (0.001>P). لم تكن مستويات الكرياتينين واليوريا مختلفة اختلافا كبيرا بين المجموعتين. وكان ترسيب كريات الدم (84.00 مم / ساعة) وتركيز البروتين الارتكاسي C (59.60 مجم / لتر) أعلى بكثير في مجموعة فيروس العوز المناعي البشري / السل عن مجموعة فيروس العوز المناعي البشري / العدوى السلية غير المتفطرة (52.00 ملم / ساعة و 19.60 مجم / لتر على التوالي)0.001>P). للتمييز بين عدوى السل عن العدوى السلية غير المتفطرة، كانت أفضل قيمة الحدية 69.5 مم / ساعة لترسيب كريات الدم، مع قيمة تنبؤية إيجابية (0.740 PPV) وقيمة تنبؤية سلبية (NPV) من 0.721، و 48.8 مجم / لتر للبروتين الارتكاسي C، مع قيمة تنبؤية إيجابية 0.676 وقيمة تنبؤية سلبية 0.697.

استنتاج: يمكن أن يساعد طابع النشر وكذلك الاستجابة المناعية القوية التي تتميز بعلامات التهاب أعلى (مثل خلايا الدم البيضاء، ترسيب كريات الدم، البروتين الارتكاسي C) على تمييز السل عن العدوى السلية غير المتفطرة في المرضى المصابين بعدوى فيروس العوز المناعي البشري الذين يحتاجون إلى العلاج التجريبي أو العلاج التشخيصي على الفور في المناطق ذات الدخل المنخفض.

Translated from English version into Arabic by Mahmoud Sami, through

TRANSLATORS WITHOUT BORDERS

常规检测指标用于鉴别 HIV 合并结核分支杆菌和非结核分枝杆菌感染

蔡仁田，于凤雪，陶臻，钱雪琴，陈军，卢洪洲

摘要
背景：HIV 人群合并结核分支杆菌（TB）和非结核分枝杆菌（non-tuberculous mycobacterial，NTM）感染多见，且临床表现非常类似，但是治疗有区别，预后有差异。临床医生有时候需要对疑似患者的治疗方法做出快速选择，然而迅速鉴别 HIV/TB 和 HIV/NTM 感染很难，尤其是在发展中国家。使用常规血液检测指标这一简单的方法区分两种感染的不同特点是很有意义的。
方法：收集自 2010 年 5 月-2016 年 3 月首次经过分支杆菌培养和菌种鉴定的 HIV/TB 和 HIV/NTM 感染者资料，进行回顾性队列分析。这些资料包括血常规，肝功能，肾功能，C 反应蛋白（CRP）和红细胞沉降率（ESR），比较两组间表达差异。
结果：总共收集 240 例合格患者，其中 HIV/TB 和 HIV/NTM 感染者分别有 113 和 127 例。两组间 CD4 T 细胞计数，年龄，性别和在确诊 TB 和 NTM 感染前的起始抗病毒患者比例没有明显差异。NTM 感染病灶更可能集中在肺部，而 TB 感染部位更加广泛，包括肺部和肺外。HIV/TB 组的白细胞计数(5.60 [4.00 – 7.85] × 10^9/L)和中性粒细胞占白细胞比例(76.70%[66.50 – 85.60%])均高于 HIV/NTM 组(4.40 [3.30 – 6.30] × 10^9/L [P=0.0014] 和 69.30% [59.10 –80.10%] [$P < 0.001$])。肝功能标志物白蛋白浓度在 HIV/TB 组中要明显低于 HIV/NTM 组($P < 0.001$)，而谷丙转氨酶（alanine aminotransferase，ALT）和谷草转氨酶（aspartate aminotransferase，AST）在两组间无明显差异。肾功能标志物肌酐和尿素氮在两组间也没有明显差异。炎性标志物 CRP 和 ESR 在 HIV/TB 组中要明显高于 HIV/NTM 组($P < 0.001$)。为了区分 TB 和 NTM 感染，ESR 最佳 cut-off 值为 69.5 mm/h，阳性预测值为 0.740 和阴性预测值为 0.721。而 CRP 的最佳 cut-off 值为 48.8 mg/L，阳性和阴性预测值分别为 0.676 和 0.697。
结论：HIV 感染者中，TB 具有更强的播散能力以及更强的炎症反应（例如白细胞计数, ESR，CRP），这些特征可以帮助低收入地区医生及时鉴别 NTM 感染，并对患者进行经验性或者诊断性治疗。

Translated from English version into Chinese by Ren-tian Cai

Valeur prédictive d'indicateurs habituellement détectés dans le plasma pour l'identification des patients infectés par le VIH porteurs d'infections mycobactériennes non tuberculeuses et tuberculeuses

Ren-tian Cai, Feng-xue Yu, Zhen Tao, Xue-qin Qian, Jun Chen, Hong-zhou Lu

Résumé
Contexte: Il est difficile de distinguer au premier abord les infections mycobactériennes non tuberculeuses (IMNT) de la tuberculose (TB) chez les patients infectés par le virus de l'immunodéficience humaine (VIH), en raison des nombreuses similitudes entre ces infections. Les pays en voie de développement ont besoin d'un moyen simple et efficace de les différencier au moyen d'analyses de sang de routine.
Méthodes: Une étude de cohortes rétrospective a été menée dans le but de recruter des patients infectés par le VIH et porteurs soit d'une IMNT, soit de la tuberculose, dont le diagnostic initial a été posé par culture des mycobactéries et identification au microscope entre mai 2010 et mars 2016. Les données incluent l'analyse des hématies, la fonction hépatique et rénale, la protéine C-réactive (PCR) et le la

vitesse de sédimentation des érythrocytes (VS). Elles ont été comparées entre les groupes VIH/TB et VIH/MNT.

Résultats: Au total, 420 patients ont été recrutés : 113 dans le groupe VIH/TB et 127 dans le groupe VIH/MNT. Aucune différence significative n'a été relevée pour la numération des CD4, l'âge, le sexe et le pourcentage de patients ayant commencé un traitement antirétroviral (ARV) avant le diagnostic explicite de tuberculose ou d'IMNT. Les IMNT sont plus souvent circonscrites aux poumons, tandis que la tuberculose affecte aussi des sites extrapulmonaires. La numération leucocytaire (5,60 × 109/l) et la proportion de neutrophiles dans celle-ci (76,70 %) étaient significativement plus élevées dans le groupe VIH/TB que dans le groupe VIH/MNT (4,40 × 109/l [*P*=0,0014] et 69,30 % [*P*<0,01]. L'analyse des marqueurs de la fonction hépatique a indiqué que la concentration d'albumine, mais pas l'ALAT ni l'ASAT, était significativement plus basse dans le groupe VIH/TB que dans le groupe VIH/MNT (*P*<0,001). Les taux de créatinine et d'urée n'étaient pas significativement différents entre les deux groupes. La VS (84,00 mm/heure) et la concentration de PCR (59,60 mg/l) étaient significativement plus élevées dans le groupe VIH/TB que dans le groupe VIH/MNT (52,00 mm/heure et 49,60 mg/l respectivement) (*P*<0,001). Pour distinguer la tuberculose des IMNT, la meilleure valeur de discrimination était une VS de 69,5 mm/heure, qui a une valeur prédictive positive (VPP) de 0,740 et une valeur prédictive négative (VPN) de 0,721, et une PCR à 48,8 mg/l, avec une VPP de 0,676 et une VPN de 0,697.

Conclusion: Le caractère disséminé et la réponse immunitaire plus forte, caractérisée par des marqueurs d'inflammation plus élevés (par ex. leucocytes, VS, PCR) peuvent aider à différencier la tuberculose des IMNT chez les patients infectés par leVIH qui ont besoin d'un traitement empirique ou d'un traitement diagnostique immédiat dans les régions à faibles revenus.

Translated from English version into French by Suzanne Assenat, through

TRANSLATORS
WITHOUT BORDERS

Обнаруживаемые на регулярной основе индикаторы в плазме обладают прогностическим потенциалом при выявлении ВИЧ-инфицированных пациентов как с нетуберкулезными микобактериальными, так и с туберкулезными инфекциями

Рентянь Цай, Фэн-Сюэ Ю, Чжэнь Тао, Сюэ-цинь Цянь, Юнь Чэнь, Хон-чжоу Лу

Реферат

Справочная информация: Оперативное выявление различий между нетуберкулезными микробактериальными (НТМ) и туберкулезными (ТБ) инфекциями в случаях с вирусом иммунодефицита человека (ВИЧ)-инфецированных пациентов преставляет определенную сложность вследствие значительного сходства двух означенных заболеваний. Для выявления указанных различий требуется стандартный анализ крови, который следует применять в развивающихся странах.

Методы: Было проведено ретроспективное когортное исследование, в котором приняли участие ВИЧ-инфецированные пациенты как с НТМ, так и с ТБ инфекциями, которым в период с мая 2010 года по март 2016 года впервые была произведена диагностика с использованиеммикобактериальных культур и микроскопического определения. По указанным данным, включающим анализ кровяных клеток, печёночной и почечной функций, С-реактивного белка (СРБ) и скорости оседания эритроцитов (СОЭ) (ЭПР), было проведено сравнение между ВИЧ/ТБ-инфецированными и ВИЧ/НТМ-инфецированными группами.

Результаты: В исследовании приняло участие в общей сложности 240 пациентов. Число ВИЧ/ТБ-инфецированных и ВИЧ/НТМ-инфецированных пациентов составляли 113 и 127 соответственно. По количеству Т-клеток CD4, возрасту, половой принадлежности, а также по процентному соотношению пациентов, начавших курс высокоактивной антиретровирусной терапии (ВААРТ) до прямой диагностики ТБ или ТНМ инфекций не наблюдалось существенных различий. НТМ инфекции с гораздо большей вероятностью ограничивались областью лёгких, тогда как ТБ инфекции обладали способностью распространяться за пределы лёгочной системы. Как число лейкоцитов(5.60 × 10^9/L), так и содержание нейтрофилов в лейкоцитах (76.70%) у группы ВИЧ/ТБ были значительно выше, чем аналогичные показатели у группы ВИЧ/НТМ (4.40 × 10^9/L [P=0.0014] и 69.30% [P<0.001]. Анализ индикаторов печёночной функции указал на более низкую концентрацию альбумина в группе ВИЧ/ТБ, чем в группе ВИЧ/НТМ (P<0.001), что однако не повлияло на показатели АлАт и АсАт. Уровень креатинина и мочевины между двумя группами существенно не различался. СОЭ (84.00 мм/час) и концентрация СРБ (59.60 мг/л) были значительно выше в группе ВИЧ/ТБ, чем в групппетВИЧ/НТМ (52.00 мм/час и 19.60 мг/л соответственно) (P< 0,001). Оптимальное значение для выявления разницы между ТБ и НТМ инфекциями составило 69.5 мм/час по СОЭ со значением положительного прогнозтического потенциала (PPV) в 0.740 и отрицательного прогностического потенциала (NPV) в 0.721, а также 48.8 мг/л по СРБ с PPV равным 0.676 и NPV составляющим 0.697.

Заключение: Характер распространения наряду с более сильной иммунной реакцией, характеризующийся более высокими показателями индикаторов воспаления (например, лейкоцитов, СОЭ, СРБ), способен содействовать выявлению разницы между ТБ и НТМ инфекциями у ВИЧ-инфицированных больных, проживающих в районах с низкими доходами и нуждающихся в проведении эмпирической или диагностической терапии.

Translated from English version into Russian by Liudmila Tomanek (nee Volynets), through

TRANSLATORS
WITHOUT BORDERS

Los marcadores detectados en los análisis sistemáticos de plasma tienen un efecto predictivo sobre el diagnóstico de la tuberculosis y las infecciones por micobacterias no tuberculosas en los pacientes infectados por el VIH

Ren-tian Cai, Feng-xue Yu, Zhen Tao, Xue-qin Qian, Jun Chen, Hong-zhou Lu

Resumen

Introducción: Es difícil diferenciar rápidamente la tuberculosis (TB) de las infecciones causadas por micobacterias no tuberculosas (MNT) en pacientes infectados por el VIH debido a las numerosas similitudes que existen entre ambas enfermedades. Los países en vías de desarrollo deberían disponer de un método de caracterización simple y eficaz que emplease los análisis sistemáticos de sangre.

Materiales y métodos: Se llevó a cabo un estudio de cohortes retrospectivo para seleccionar pacientes infectados por el VIH con TB o con infección por MNT diagnosticados por primera vez entre mayo de 2010 y marzo de 2016 mediante cultivo e identificación microscópica de las micobacterias. Los datos obtenidos incluían: el análisis de las células sanguíneas, la función hepática y renal, los valores de la proteína C reactiva (PCR) y la velocidad de sedimentación globular (VSG). Los resultados de estas determinaciones se compararon entre los grupos con VIH/TB y con VIH/MNT.

Resultados: Se inscribieron un total de 240 enfermos: 113 con HIV/TB y 127 con VIH/MNT. No se hallaron diferencias significativas previas al diagnóstico de TB o de infección por MNT en el recuento de linfocitos CD4, en la edad, en el sexo, ni en el porcentaje de pacientes que acababan de comenzar la terapia antirretroviral (TAR). La mayoría de las infecciones por MNT se localizaron en los pulmones, mientras que la TB se diseminó también a otros órganos. Tanto el recuento total de leucocitos ($5{,}60 \cdot 10^9$/L) como la proporción de neutrófilos de la fórmula leucocitaria (76,70 %) era significativamente mayor en el grupo con VIH/TB que en el grupo con VIH/MNT ($4{,}40 \cdot 10^9$/L [$P = 0{,}0014$] y 69,30 % [$P < 0{,}001$]). El análisis de los marcadores de la función hepática mostró que la concentración de albúmina era significativamente menor en el grupo con HIV/TB que en el grupo con VIH/MNT ($P < 0{,}001$), pero no así la actividad de la ALT y la AST. No se observaron diferencias significativas entre las concentraciones de creatinina y urea de ambos grupos. La VSG (84,00 mm/h) y la concentración de la PCR (59,60 mg/L) del grupo con VIH/TB eran significativamente superiores a las del grupo con VIH/MNT (52,00 mm/h y 19,60 mg/L, respectivamente) ($P < 0{,}001$). Para diferenciar la tuberculosis de las infecciones por MNT, el valor de corte más apropiado para la VSG fue 69,5 mm/h, con un valor predictivo positivo (VPP) de 0,740 y un valor predictivo negativo (VPN) de 0,721, mientras que para la PCR fue de 48,8 mg/L con un VPP de 0,676 y un VPN de 0,697.

Conclusiones: Su tendencia a la diseminación, así como la reacción inmunitaria que provoca, más intensa y con valores superiores de los marcadores de inflamación (p. ej., RTL, VSG y PCR), pueden servir para diferenciar la TB de la infección por MNT en pacientes infectados por el VIH que requieran diagnóstico o tratamiento empírico inmediato en las regiones con menor nivel adquisitivo.

Translated from English version into Spanish by B_A_Iturregui, through

TRANSLATORS
WITHOUT BORDERS
